# Oral Medications Enhance Adherence to Surveillance for Hepatocellular Carcinoma and Survival in Chronic Hepatitis B Patients

**DOI:** 10.1371/journal.pone.0166188

**Published:** 2017-01-18

**Authors:** Joon Yeul Nam, Jeong-Hoon Lee, Hwi Young Kim, Jieun E. Kim, Dong Hyeon Lee, Young Chang, Hyeki Cho, Jeong-Ju Yoo, Minjong Lee, Young Youn Cho, Yuri Cho, EunJu Cho, Su Jong Yu, Yoon Jun Kim, Jung-Hwan Yoon

**Affiliations:** 1 Department of Internal Medicine and Liver Research Institute, Seoul National University College of Medicine, Seoul, Korea; 2 Department of Internal Medicine and Liver Center, Ewha Womans University School of Medicine Seoul, Korea; 3 Department of Brain and Cognitive Sciences, Ewha Womans University, Seoul, Korea; 4 Department of Internal Medicine, Seoul Metropolitan Government Seoul National University Boramae Medical Center Seoul, Korea; 5 Digestive Disease Center and Research Institute Department of Gastroenterology and Hepatology, Soonchunhyang University Bucheon Hospital; 6 Department of Internal Medicine, Kangwon National University Hospital, Chuncheon, South Korea; 7 Department of Internal Medicine CHA Gangnam Medical Center, CHA University; National Taiwan University College of Medicine, TAIWAN

## Abstract

**Background/Aims:**

Regular surveillance for hepatocellular carcinoma (HCC) in chronic hepatitis B (CHB) patients is essential to detect HCC earlier and to improve prognosis. This study investigated whether prescription of oral medication contributes to adherence to surveillance, early tumor detection, and overall survival (OS).

**Methods:**

A total of 401 CHB patients who were newly diagnosed with HCC were included: 134 patients received no medication (group 1), 151 received hepatoprotective agents such as ursodeoxycholic acid and silymarin (group 2), and 116 received antiviral agents (group 3) at two years before HCC diagnosis. The primary endpoint was OS, and secondary endpoints were compliance to regular surveillance and HCC status at diagnosis.

**Results:**

Compared to group 1, both group 2 and 3 had higher rates of good compliance to regular surveillance (defined as participation in >80% of imaging intervals being ≤6 months) (58.2%, 90.1%, and 97.4%, respectively; *P*<0.001), more HCC diagnosed at a very early stage (20.9%, 32.5%, and 36.2%; *P* = 0.019) and smaller tumor size (2.8±2.4cm, 1.9±1.1cm, and 1.8±0.9cm; *P*<0.001). Finally, compared to group 1, both group 2 (hazard ratio, 0.63; 95% confidence interval, 0.41–0.97; *P* = 0.035) and group 3 (hazard ratio, 0.40; 95% confidence interval, 0.22–0.71; *P* = 0.002) had significantly longer OS. In mediation analysis, prolonged OS is resulted considerably from indirect effect mediated by shorter imaging interval (>100% in group 2 and 14.5% in group 3) rather than direct effect of medication itself.

**Conclusions:**

Prescription of oral medication improves compliance to surveillance and enables early detection of HCC, which is associated with enhanced survival.

## Introduction

World-wide, hepatocellular carcinoma (HCC) is the fifth most common cancer in men and the seventh most common in women. HCC seldom develops in the absence of risk factors, such as chronic hepatitis B virus (HBV) or hepatitis C virus (HCV) infection, alcoholic liver cirrhosis, nonalcoholic steatohepatitis, or aflatoxin exposure.[1] Thus, theoretically, regular surveillance for HCC in patients with those risk factors is essential to detect HCC earlier and to improve prognosis.[2]

International guidelines recommend regular surveillance for chronic hepatitis patients with ultrasonography (US) or multi-phase computed tomography (CT) at 4–6 month intervals.[2–4] In practice, patients with good compliance to regular surveillance have HCC detected at earlier stages than patients with poor compliance. Furthermore, early detection of HCC is linked to improved overall survival (OS).[5] However, a considerable number of patients at risk are not aware of the importance of regular surveillance. This poor adherence to HCC surveillance leads to detection at advanced stages, drastically lowering OS.[6]

The health belief model (HBM) is a systematic method to predict preventive health behavior, which was proposed in the 1950s by social psychologists to understand challenges in screening and follow-up for tuberculosis.[7] HBM has been applied to predict health-related behaviors, such as receiving immunizations for infectious disease and undergoing screening for asymptomatic diseases like early stage cancer.[8] It encompasses the relationship of health behaviors, practices, utilization of services, and general health motivation.[9] Chronic hepatitis patients who are asymptomatic may not perceive the importance of regular surveillance for their disease, leading to irregular or no surveillance. This behavioral tendency can be explained by HBM. Instilling good compliance based on HBM has been accomplished in many chronic diseases, including diabetes mellitus, asthma, and coronary heart disease; an approach of increasing perceived disease severity worked well and resulted in good prognosis.[10–12]

In this study, prescription of oral medication (i.e., hepatoprotective agents or antiviral agents) was used to increase perceived disease severity.[13–15] We evaluated whether prescriptions contributed to early tumor detection and improved survival in patients with chronic hepatitis B, which is the most common cause of HCC.

## Methods

### Patients

This retrospective study screened inpatient and outpatient database files at a single tertiary hospital (Seoul National University Hospital; Seoul, Korea) between January 1, 2007 and December 31, 2012 to select a cohort of consecutive adult patients who were diagnosed with HBV-related HCC. All subjects were followed as chronic hepatitis B (CHB) patients for at least two years before diagnosis of HCC. HCC diagnosis was based on the guidelines of the American Association for the Study of Liver Diseases.[2, 16] Patients were excluded if they met any of the following criteria: age <18 years; co-infection with other hepatotrophic viruses (i.e., hepatitis C or D virus) or human immunodeficiency virus; other previous or current malignancies except for HCC; or severe comorbidities, such as chronic kidney disease, chronic obstructive pulmonary disease, or cardiac diseases.

This study complied with the Declaration of Helsinki. The study protocol was approved by the Institutional Review Board of Seoul National University Hospital, and the requirement for informed consent from patients was waived.

### Study design

All patients were educated about the significance of attending regular follow-up appointments, at least every 6 months with alpha-fetoprotein (AFP) level measurement and imaging.[4, 17] Patients were categorized into 3 groups according to the treatment they received for 2 years before the diagnosis of HCC: no medication (group 1), hepatoprotective agents (group 2) or antiviral agents (group 3). Patients who were followed with medication other than hepatoprotective or antiviral agents were planned to be classified as hepatoprotective group (group 2). The criteria for antiviral treatment of enrolled patients followed the Korean National Healthcare Insurance (a single player insurance system in Korean) coverage criteria which are mainly based on AASLD guidelines. After the HCC diagnosis, most patients could be treated with antiviral therapy if serum HBV DNA was detectable in accordance with the national insurance criteria. If a patient switched treatment from no medication or a hepatoprotective agent to an antiviral more than 1 year before the HCC diagnosis, the patient was considered part of the antiviral group, whereas patients who switched less than 1 year before the HCC diagnosis were excluded. The primary endpoint was OS, measured from the date of HCC diagnosis to death from any cause. Survival status of all patients was evaluated based on national statistical data obtained from the Korean Ministry of Government Administration and Home Affairs. Secondary endpoints were compliance to regular surveillance and stage of HCC at diagnosis. Regular surveillance was defined as having >80% of CT or US imaging with intervals ≤ 6 months.[18–20]

Patient characteristics collected at the time of HCC diagnosis included: age, sex, body mass index, performance status according to Eastern Cooperative Oncology Group (ECOG), diabetes mellitus, hypertension, laboratory findings, Child-Turcotte-Pugh (CTP) class, model for end-stage liver disease (MELD) score, presence of cirrhosis, and year of HCC diagnosis. Clinical diagnosis of cirrhosis was determined as follows: (i) platelet count of <100,000/mL and US findings suggestive of cirrhosis, including a blunted, nodular liver edge accompanied by splenomegaly (>12 cm) or (ii) clinical signs of portal hypertension, such as ascites, esophageal or gastric varices, and hepatic encephalopathy.[21, 22] Initial HCC stage was evaluated by Barcelona Clinic Liver Cancer (BCLC) staging system. Data on the following tumor characteristics were also collected: tumor type (nodular, infiltrative, massive, or diffuse), multiplicity of tumor, maximum size of the largest tumor, vascular invasion, portal vein tumor thrombosis, adjacent organ invasion, and distance metastasis.

### Statistical analysis

Continuous data are expressed as mean ± standard deviation or frequency (percent) and discrete variables as absolute and relative frequencies. One-way ANOVA and Student t-test were applied to compare continuous data. Categorical variables were compared with Pearson’s Chi-square tests. OS was calculated as the time from HCC diagnosis until death from any cause. Possible predictors for OS were evaluated by univariate Cox regression analysis. Factors with a P value < 0.05 in univariate analyses were included in multivariate Cox regression and survival analyses. To verify the effect of regular surveillance on OS, mediation analysis was performed. Imaging intervals of each group were designated as mediators. With this mediator, direct effect (pharmacological effect) and indirect effect (non-pharmacological effect) of medications on OS was calculated. Mediation analysis with survival date was performed using accelerated failure time model, based on previous report.[23, 24] To adjust for the lead-time bias, we utilized Schwartz’s equation.[25] The doubling time of HCC is reported to be approximately 120–180 days;[26] therefore, we used 180 days as the most conservative HCC doubling time. Statistical analysis was performed by SPSS version 21 (IBM) and SAS 9.3 (SAS Institute).

## Results

### Patient characteristics

**Fig 1:**
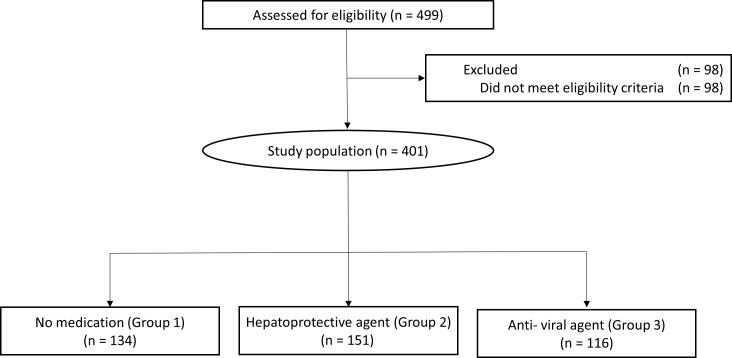
CONSORT diagram. A total of 499 HCC patients were identified of whom 98 did not meet inclusion criteria. The final sample consisted of 401 patients, who were classified into three groups according to prescription of oral medication.

As shown in Fig 1, a total of 499 patients were identified through database screening; of whom 98 were excluded because of other malignancies or severe comorbidities such as chronic kidney disease, chronic obstructive pulmonary disease, and cardiac disease. Among the final sample of 401 patients, 134 were followed up without any medication (designated as group 1), 151 with hepatoprotective agents (group 2) and 116 with antiviral agents (group 3) until the time of HCC diagnosis. All patients who received medications for cirrhotic complications (such as lactulose, beta blocker, or lactulose) also received either hepatoprotective agents or antiviral agents. After diagnosis of HCC, oral antiviral treatment was done mostly following practice guidelines from European Association for the Study of the Liver. [27]

As shown in Table 1, at the time of HCC diagnosis, group differences were observed for tumor factors, serum albumin levels (*P* = 0.023), and the year of HCC diagnosis (*P* = 0.035). CTP class (*P =* 0.761), MELD score (*P =* 0.676), and proportion of cirrhosis at the time of HCC diagnosis were similar across groups (*P =* 0.450).

**Table 1:** Patients' characteristics at the time of HCC diagnosis.

	Parameter	Total (%)	Group 1	Group 2	Group 3	*P-*value
		(*n* = 401)	(*n* = 134)	(*n* = 151)	(*n* = 116)	
Age, mean±SD, years		59.4±8.1	58.0±8.3	59.8±8.1	60.4±7.8	0.051‡
Sex, N (%)	Male	308 (76.8%)	108 (80.6%)	122 (80.8%)	87 (75.0%)	0.150§
BMI mean±SD, m^2^/kg		23.5±3.3	23.1±3.1	23.5±3.4	24.1±3.3	0.073§
ECOG*, N (%)	0	158 (39.4%)	64 (47.8%)	45 (29.8%)	49 (42.2%)	0.881§
	1	52 (13.0%)	22 (16.4%)	13 (8.6%)	17 (14.7%)	
	≥2	12 (3.0%)	2 (1.5%)	4 (2.6%)	6 (5.2%)	
	Missing	179 (44.6%)	46 (34.3%)	89 (58.9%)	44 (37.9%)	
Diabetes, N (%)		71 (17.7%)	21 (15.7%)	26 (17%)	24 (21%)	0.573§
Hypertension, N (%)		86 (21.4%)	29 (21.6%)	27 (17.9%)	30 (25.9%)	0.289§
Platelet, ×10^3^/mm^3^		115.9±52.7	118.2±41.4	114.9±62.1	114.8±51.4	0.837‡
Albumin, g/dL		3.9±0.5	3.9±0.5	3.8±0.6	4.0±0.5	0.023‡
Total bilirubin, mg/dL		1.4±2.3	1.5±3.3	1.3±0.8	1.4±2.2	0.852‡
ALP, IU/L		92.8±45.3	97.6±59.6	90.1±33.0	90.8±39.3	0.318‡
AST, IU/L		48.2±48.0	48.0±35.9	48.5±25.3	48.1±75.3	0.996‡
ALT, IU/L		47.4±45.8	48.0±37.3	47.6±29.3	46.6±67.5	0.967‡
PT INR		1.14±0.16	1.14±0.20	1.14±0.13	1.13±0.16	0.749‡
HBeAg-positive, N (%)		58 (22.8%)	17 (25.4%)	16 (16.0%)	25 (28.7%)	0.100‡
AFP, ng/mL		326.7±1987.7	449.9±1929.5	380.8±2647.8	115.2±472.2	0.381‡
MELD score, mean±SD		9±3.5	9±3.7	9±3.6	9±3.3	0.676‡
CTP class, N (%)	A	352 (87.8%)	116 (86.6%)	132 (87.4%)	104 (89.7%)	0.761§
	B	45 (11.2%)	16 (11.9%)	17 (11.3%)	12 (10.3%)	
	C	4 (1.0%)	2 (1.5%)	2 (1.6%)	0 (0.0%)	
Liver cirrhosis^†^, N (%)		122 (90.0%)	138 (91.0%)	101 (91.4%)	376 (87.1%)	0.450§
Year of HCC diagnosis,	2007	57 (14.2%)	17 (12.7%)	29 (19.2%)	11 (9.5%)	0.035∥
N (%)	2008	54 (13.5%)	22 (16.4%)	22 (14.6%)	10 (8.6%)	
	2009	63 (15.7%)	23 (17.2%)	25 (16.6%)	15 (12.9%)	
	2010	56 (14.0%)	11 (8.2%)	34 (22.5%)	11 (9.5%)	
	2011	45 (11.2%)	13 (9.7%)	16 (10.6%)	16 (13.8%)	
	2012	126 (31.4%)	48 (35.8%)	25 (16.6%)	53 (45.7%)	

BMI, body mass index; ECOG, Eastern Cooperative Oncology Group; ALP, alkaline phosphatase; AST, aspartate transaminase; ALT, alanine transaminase; PT INR, prothrombin time international normalized ratio; AFP, alpha-fetoprotein; MELD, Model for End-stage Liver Disease; CTP, Child-Turcotte-Pugh.

Note. Data are expressed as n (%) or mean±SD.

* The ECOG performance status assesses on a scale ranging from 0 (fully active) to 5 (dead).

† Liver cirrhosis was diagnosed by the presence of histological and radiological evidence.

‡ by One-way ANOVA.

§ by Pearson's Chi-square test.

∥ by Linear-by-linear association test.

### Compliance to surveillance

As shown in Table 2, the proportion of good compliance to regular surveillance (defined as > 80% of CT or US intervals being ≤ 6 months) was significantly higher in patients receiving oral medications [both groups 2 (90.1%) and 3 (97.4%)] than in patients not receiving any medication [group 1 (58.2%)] (both P < 0.001) (S1 Fig). The proportion of good compliance was also significantly higher in group 3 than group 2 (P = 0.018). The median intervals of US or CT imaging were significantly different among groups. Group 1 showed a significantly longer interval (median, 6.0 months; range, 4.0–24.0 months) than both group 2 (median, 6.0 months; range, 3.0–12.0) and group 3 (median, 5.0 months; range, 3.0–8.0) (both P < 0.001).

**Table 2:** Follow-up interval of patients.

Median follow-up interval	Total (*n* = 401)	Group 1 (*n* = 134)	Group 2 (*n* = 151)	Group 3 (*n* = 116)	*P*-value (1 *vs*. 2)	*P*-value (1 *vs*. 3)	*P*-value *(2 vs*. 3)	*P*-value (1 *vs*. 2+3)
Imaging interval (months)	6.0 (3.0–24.0)	6.0 (4.0–24.0)	6.0 (3.0–12.0)	5.0 (3.0–8.0)	< 0.001*	< 0.001*	0.004*	< 0.001*
Visit interval (months)	4.8(3.0–24.0)	6.0(3.0–24.0)	4.0(3.0–12.0)	4.0(3.0–6.0)	< 0.001*	< 0.001*	0.002*	< 0.001*
**Surveillance**	**Total (*n* = 401)**	**Group 1 (*n* = 134)**	**Group 2 (*n* = 151)**	**Group 3 (*n* = 116)**	***P*-value (1 *vs*. 2)**	***P*-value (1 *vs*. 3)**	***P*-value *(2 vs*. 3)**	***P*-value (1 *vs*. 2+3)**
Regular surveillance	327 (81.5%)	78 (58.2%)	136 (90.1%)	113 (97.4%)	< 0.001†	< 0.001†	0.018†	< 0.001†
Irregular surveillance	74 (18.5%)	56 (41.8%)	15 (9.9%)	3 (2.6%)				

Note. Data are expressed as n (%) or median with minimum and maximum. Regular follow-up was defined as >80% of CT or US imaging intervals being ≤ 6 months.

* by Student t-test.

† by Pearson's Chi-square test.

### Tumor characteristics at the time of HCC diagnosis

Most HCCs were detected at an early stage across the treatment groups. A diffuse, infiltrative or massive HCC was diagnosed in four patients in group 1 (3.0%), but none in group 2 or 3. The percentage of single nodular tumors was smaller in group 1 than in group 2 or 3 (66.3%, 84.8%, and 84.5%, respectively, both P < 0.001). The maximal tumor size was larger in group 1 than both group 2 and 3 (2.8 ± 2.4, 1.9 ± 1.1, and 1.8 ± 0.9 cm, respectively; both P < 0.001). Major vessel invasion at the time of diagnosis was more frequent in group 1 than 2 or 3 (13.4%, 1.3%, 0.9%, respectively, both P < 0.001). The percentage of HCC diagnosed at a very early stage (BCLC stage 0) was higher in both group 2 (32.5%) and group 3 (36.2%) than in group 1 (20.9%) (both P < 0.05). The percentage diagnosed in an advanced or end stage (BCLC stage C or D) was lower in both group 2 (37.1%) and group 3 (32.8%) than in group 1 (42.5%) (both P < 0.05). Curative therapies (i.e., liver transplantation, surgical resection, radiofrequency ablation, or percutaneous ethanol injection) were more frequently administered as an initial treatment in group 2 (63.6%) and group 3 (57.7%) than in group 1 (52.3%). The same tendencies were found when the patients were categorized according to follow-up interval by 6 months (Table 3). Most HCCs were detected at earlier stages according to both TNM and BCLC staging systems when the median imaging interval of patients was < 6 months (*P* < 0.05).

**Table 3:** Initial tumor characteristics according to oral medication and follow up interval.

Tumor	Parameter	Group 1	Group 2	Group 3	*P*-value	*P*-value	*P*-value	*P*-value	Median imaging interval	Median imaging interval	*P*-value
characteristics		(*n* = 134)	(*n* = 151)	(*n* = 116)	(1 vs. 2)	(1 vs. 3)	(2 vs. 3)	(3 groups)	≤ 6 months (*n* = 309)	> 6 months (*n* = 92)	
Type	Nodular	130 (97.0%)	151 (100.0%)	116 (100.0%)	< 0.001*	< 0.001*	< 0.001*	0.018*	308 (99.7%)	89 (96.7%)	0.013*
	Diffuse/infiltrative/massive	4 (3.0%)	0 (0.0%)	0 (0.0%)					1 (0.3%)	3 (3.3%)	
Numbers	Single	89 (66.3%)	128 (84.8%)	98 (84.5%)	< 0.001*	0.004*	0.949*	< 0.001*	251 (81.2%)	64 (69.6%)	0.017*
	Multiple	45 (33.6%)	23 (15.2%)	18 (15.5%)					58 (18.8%)	28 (30.4%)	
Maximum size	cm	2.8±2.4	1.9±1.1	1.8±0.9	< 0.001†	< 0.001†	0.234†	< 0.001‡	1.9±1.1	3.1±2.7	< 0.001†
MVI		18 (13.4%)	2 (1.3%)	1 (0.9%)	< 0.001*	0.001*	0.722*	< 0.001*	12 (3.9%)	9 (9.8%)	0.026*
PVTT		19 (14.2%)	2 (1.3%)	1 (0.9%)	< 0.001*	0.001*	0.722*	< 0.001*	12 (3.9%)	9 (9.8%)	0.026*
BCLC stage	0	28 (20.9%)	49 (32.5%)	42 (36.2%)	0.048*	< 0.001*	0.811*	0.050*	102 (33.0%)	17 (18.5%)	0.012*
	A	40 (29.9%)	44 (29.1%)	34 (29.3%)					93 (30.1%)	25 (27.2%)	
	B	9 (6.7%)	2 (1.3%)	2 (1.7%)					7 (2.3%)	6 (6.5%)	
	C	54 (40.3%)	52 (34.4%)	37 (31.9%)					102 (33.0%)	42 (44.6%)	
	D	3 (2.2%)	4 (2.6%)	1 (0.9%)					5 (1.6%)	3 (3.3%)	
First treatment	Liver transplantation	2 (1.5%)	6 (4.0%)	4 (3.4%)	0.030*	< 0.001*	0.648*	0.023*	8 (2.6%)	4 (4.3%)	0.013*
modality	Surgical resection	18 (13.4%)	15 (9.9%)	10 (8.6%)					31 (10.0%)	12 (13.0%)	
	RFA	25 (18.7%)	40 (26.5%)	34 (29.3%)					81 (26.2%)	18 (19.6%)	
	PEI	25 (18.7%)	35 (23.2%)	19 (16.4%)					68 (22.0%)	11 (12.0%)	
	TACE	56 (41.8%)	54 (35.8%)	49 (42.2%)					117 (37.9%)	42 (45.7%)	
	Sorafenib	2 (1.5%)	0 (0.0%)	0 (0.0%)					9 (0.0%)	0 (2.2%)	
	Cytotoxic chemotherapy	0 (0.0%)	1 (0.7%)	0 (0.0%)					0 (0.0%)	1 (1.1%)	
	Supportive care only or lost to follow-up	6 (4.5%)	0 (0.0%)	0 (0.0%)					4 (1.3%)	2 (2.2%)	

MVI, major vessel invasion; PVTT, portal vein tumor thrombosis; BCLC, Barcelona Clinic Liver Cancer; RFA, radiofrequency ablation; PEI, percutaneous ethanol injection; TACE, transarterial chemoembolization.

Note. Data are expressed as N (%) or mean ± SD.

* by Pearson's Chi-square test.

† by Student t-test.

‡ by one-way ANOVA.

### Overall survival

Overall median follow-up duration after diagnosis of HCC was 49.0 (interquartile range, 37.0–71.0) months. During the study period, there were 43 (32.1%) deaths in group 1, 44 (29.1%) in group 2, and 16 (13.8%) in group 3.

**Fig 2:**
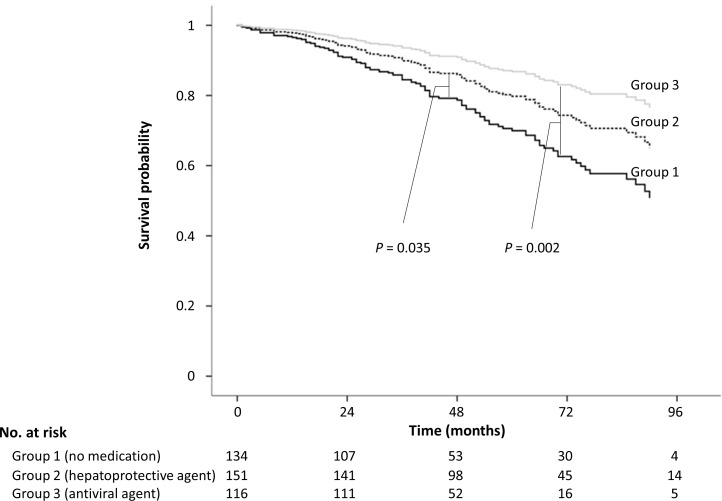
Overall survival among groups. Overall survival was highest in group 3 (antiviral agents), followed by group 2 (hepatoprotective agents), and OS was lowest in group 1 (no medication) (all *P* < 0.05).

In multivariate analysis of Cox regression, prescription of oral medication (both group 2 and 3) was an independent predictor for prolongation of OS (group 2 *vs*. group 1: HR, 0.63; 95% CI, 0.41–0.97; *P* = 0.035; group 3 *vs*. group 1: HR, 0.40; 95% CI, 0.22–0.71; *P* = 0.002) compared to no medication (group 1) after adjustment for the presence of cirrhosis and performance status (Table 4) (Fig 2). There was a subgroup of patients with good compliance to regular surveillance without medication as well in the group 1. Patients who underwent regular surveillance patients had significantly longer overall survival than those who underwent irregular surveillance in multivariate analysis in the no medication group (adjusted hazard ratio, 2.13; 95% confidence interval, 1.07–4.22; P = 0.03) (S1 Table). No factor which was associated to the surveillance compliance was found in the statistical analysis (S2 and S3 Tables).

**Table 4:** Univariate and multivariate analyses associated with overall survival.

Variables		Univariable analysis		Multivariable analysis	
		HR (95% CI)	*P*-value	HR (95% CI)	*P*-value
Age		1.018 (0.99–1.04)	0.147		
Sex	Male	1.543 (0.92–2.60)	0.103		
Cirrhosis		17.63 (2.46–126.54)	0.004	15.88 (2.21–114.12)	0.006
ECOG	0	1 (reference)	< 0.001	1 (reference)	
	1	2.19 (1.47–3.260)		2.13 (1.43–3.18)	< 0.001
	≥2	9.16 (3.86–21.76)		10.08 (4.19–24.27)	< 0.001
DM		0.98(0.57–1.61)	0.870		
HTN		1.26 (0.78–2.04)	0.352		
Year of HCC	2007	0.66 (0.24–1.81)	0.416		
diagnosis	2008	1.10(0.52–2.33)	0.807		
	2009	1.30 (0.66–2.55)	0.460		
	2010	1.47 (0.75–2.91)	0.264		
	2011	1.31 (0.66–2.58)	0.439		
	2012	1 (reference)			
Medication	Group 1*	1 (reference)		1 (reference)	
	Group 2†	0.72 (0.47–1.10)	0.124	0.63 (0.41–0.97)	0.035
	Group 3‡	0.42 (0.24–0.74)	0.003	0.40 (0.22–0.71)	0.002

HR, hazard ratio; CI, confidence interval; ECOG, Eastern Cooperative Oncology Group; DM, diabetes mellitus; HTN, hypertension.

Note. Data are expressed as n (%) or median with minimum and maximum.

* Group which is followed with no medication.

† Group which is followed with hepatoprotective agents.

‡ Group which is followed with antiviral agents.

**Fig 3:**
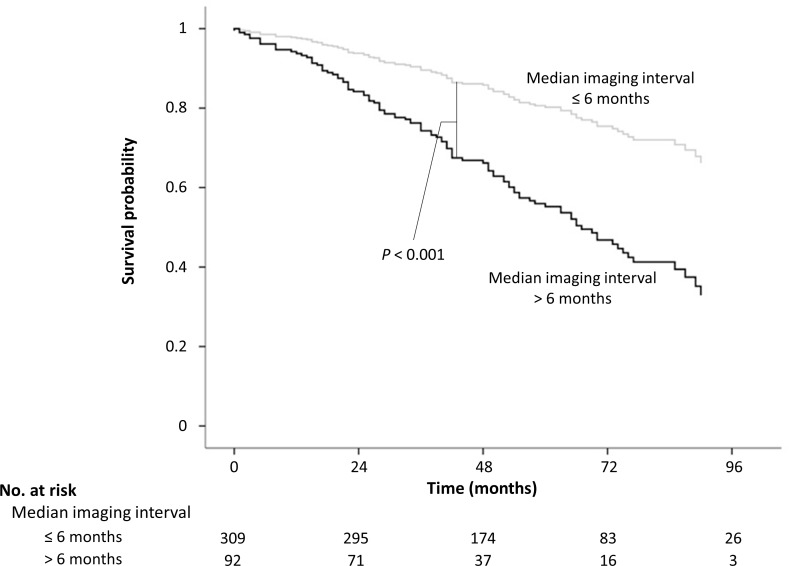
Mediation analysis. Median imaging (US or CT) interval was used as mediator in mediation analysis. Patients were divided in 2 groups by median imaging intervals. Overall survival was significantly longer in patients with median imaging interval ≤ 6 months than those with median imaging interval exceeding 6 months (*P* < 0.001).

To evaluate the direct and indirect effect on OS, mediation analysis was performed using median value of imaging interval as a mediator. In comparison between group 1 and group 2, indirect effect (i.e., shorter imaging interval in group 2) of medication accounted for approximately 100% of total effect of medication (hepatoprotective agents in group 2) on prolonged OS, which indicates that increase in OS was exclusively owing to shorter imaging interval, but not related to pharmacological effect of medications itself (S4A Table). In comparison between group 1 and group 3, 14.5% of total effect which increased OS in group 3 was owing to indirect effect (shorter imaging interval in group 3) of antiviral agents (S4B Table). Imaging interval had a significant impact on OS. Patients with median imaging interval ≤ 6 months which has significantly earlier HCC stage than patients with median imaging interval > 6 months had significantly lower risk of death (HR, 0.371; 95% CI, 0.249–0.554; *P* < 0.001) (S5 Table, Fig 3).

After we adjusted for lead-time bias [25] of the irregular follow-up group (median imaging interval > 6 months), oral medication prescription (groups 2 and 3) was still an independent predictor for OS prolongation in multivariate analysis of Cox’s regression (S6 Table). OS was significantly longer in groups 2 and 3 than group 1 (S2 Fig).

## Discussion

This study showed improved compliance to regular surveillance in patients who received prescriptions of oral medication compared to those who did not. Patients with oral medication had more regular surveillance, were diagnosed with HCC at an earlier stage, and consequently exhibited prolonged OS. Mediation analysis confirmed that indirect effect, which was postulated as a short imaging interval, considerably accounted for the increase in survival especially in patients taking hepatoprotective agents.[28]

There has been no evidence that hepatoprotective agents, such as ursodeoxycholic acid and silymarin, prevent the progression of either liver cirrhosis or HCC and thus, the prescription of these agents are not generally recommended in the context of evidence-based medicine. In this study, surprisingly, prescription of hepatoprotective agents also improved adherence of CHB patients to surveillance, resulting in earlier detection of HCC and improved OS after HCC diagnosis although the effect size was smaller compared to antiviral agents. Antiviral agents can also enhance the specificity of AFP by reducing false-positive results, which can help in the early detection of HCC.[29] From these findings, it may be speculated that OS in patients taking hepatoprotective agents (group 2) might be prolonged due to enhanced adherence to regular surveillance of HCC rather than the pharmacological effect of hepatoprotective drugs itself. As expected, mediation analysis supported that the increase in OS in group 2 was exclusively related to indirect effect of medication, not to direct pharmacological effect. Antiviral agents have previously been confirmed to increase survival and reduce liver cirrhosis or HCC for patients with CHB which is the most common cause of HCC.[28] In line with this preexisting literature, patients taking antiviral agents (group 3) showed the longest OS in the current study. In mediation analysis, actually, direct effect was more prominent than indirect effect on OS, which indicates antiviral agents directly prolonged OS. However, interstingly, there was a partial indirect effect also in this group. These findings collectively indicates that increase in OS of patients who were prescribed oral medications is mediated by indirect effect (i.e., the increased adherence to regular surveillance).

There might be a lead-time bias due to different surveillance intervals. Lead-time bias is usually adjusted in analyses of non-surveillance groups. However, the irregular surveillance group also has a partial lead-time bias, which may be shorter than that of the non-surveillance group. In this study, we conservatively selected 180 days as an HCC doubling time to calculate lead-time bias. The results of adjustment for longer lead-time bias according to non-surveillance and maximal HCC doubling time indicate that oral medication prescription still significantly improves OS.

The HBM is a psychological model that attempts to explain and predict health behaviors based on attitudes and beliefs of individuals. The HBM consists of six constructs: (i) perceived susceptibility (one’s opinion of chances of getting a condition), (ii) perceived severity (one’s opinion of how serious a condition and its consequences are), (iii) perceived benefits (one’s belief in the efficacy of the advised action to reduce risk or seriousness of impact), (iv) perceived barriers (one’s opinion of the tangible and psychological costs of the advised action), (v) cues to action (strategies to activate readiness), and (vi) self-efficacy (confidence in one’s ability to take action)[9] The HBM can explain how oral medication improves compliance to surveillance. Although physicians emphasize the necessity of regular surveillance to every CHB patient, a number of patients are unlikely to adhere to their recommended surveillance schedule because they are asymptomatic unless they have active hepatitis or decompensated liver function. Prescription of oral medication may impel CHB patients to take their disease more seriously, improving adherence to surveillance. According to the Korean National Cancer Screening survey in 2003, HCC surveillance rate was around 23%, and rates have decreased over time.[30, 31] Based on HBM, this tendency might be related to low perceived disease severity or low perceived susceptibility to subsequently developing HCC. Prescription of oral medication has been reported to increase perceived disease severity in hypertension patients as well.[13–15] In addition, medication can trigger a “cue to action” because regular medication may repeatedly call attention to one's medical condition that requires routine surveillance for favorable prognosis.[32] Taking medication can induce high “self-efficacy” since it may foster a sense of control over the disease or promote confidence in one’s ability to improve the prognosis with medications.[14]

International guidelines recommend regular surveillance for chronic hepatitis patients who are at risk of HCC with US or CT imaging at least every 4–6 months.[2, 3] Among CHB patients, lifetime risk of developing HCC is as high as 25% for women and 35% for men.[33] The 4–6 month surveillance interval is based on the doubling time of HCC and is recommended by most international guidelines.[34] In a previous study, surveillance at less than 3-month intervals did not improve detection of small HCC compared to 6-month intervals.[35] If HCC is detected at early stages, there is a chance of curative treatment, as shown in the current study. The percentage of curative therapies as an initial treatment was lower in group 1 than in both group 2 and group 3. More patients in group 1 were treated with transarterial chemoembolization, or cytotoxic chemotherapy, which are seldom performed with a curative intent. Two of the HCC patients in group 1 could not be treated and received only supportive care because of large HCC burden and poor liver function. These results came from the differences of compliance to surveillance protocols among groups. Although these results describe our observations, we do not recommend that physicians prescribe hepatoprotective agents to increase patient adherence to HCC surveillance protocols.

This study has a few limitations. First, subjects were only CHB patients. Further research is warranted on chronic hepatitis patients with other etiologies, such as HCV or alcohol abuse. Second, this was a retrospective study. In some ways, however, this could also be a strength because in a prospective design, informed consent which is not used in real-life clinical practice might arouse patients’ awareness of disease severity, affect their adherence to surveillance, and consequently lead to a bias. Third, education and counseling which might also increase perceived disease severity were not precisely evaluated in this study due to a retrospective design. However, an effective education program or a well-designed alarm program using text messaging or e-mail may be a better way to enhance compliance.[36, 37] Fourth, there is no quantitative measurement to evaluate patients’ health beliefs, which could be evaluated with a questionnaire. However, this was not possible to perform for this retrospective study.

In conclusion, the surveillance adherence of patients not receiving any medications is lower than those of receiving medications, regardless of pharmacologic effects. Consequently, it enables earlier detection of HCC and improves survival in chronic hepatitis B patients.

## Supporting Information

S1 Fig The cumulated number of patients among groups arranged with imaging interval (months). More patients in group 2 (hepatoprotective agents) or 3 (antiviral agent) tended to be followed up at short term imaging interval than patients of group 1 (no medication). Several patients in group 1 had a long term imaging interval which meant poor compliance to surveillance.
(TIF)

S2 Fig Overall survival among groups after an adjustment for lead-time bias. Overall survival was also highest in group 3 (antiviral agents), followed by group 2 (hepatoprotective agents), and OS was lowest in group 1 (no medication) after an adjustment of lead-time bias (all *P* < 0.05).
(TIF)

S1 Table Univariate and multivariate analyses associated with overall survival according to surveillance in no medication group. (DOCX)

S2 Table Patients' characteristics at the time of HCC diagnosis according to surveillance within no medication group. (DOCX)

S3 Table Initial tumor characteristics according to surveillance in no mediation group. (DOCX)

S4 Table Results of mediation analysis. (DOCX)

S5 Table Univariate and multivariate analyses associated with overall survival according to median follow-up interval. (DOCX)

S6 Table Univariate and multivariate analyses associated with overall survival after adjustment for lead-time bias. (DOCX)

## Abbreviations

HCC: hepatocellular carcinoma
CHB: chronic hepatitis B
OS: overall survival
HBV: hepatitis B virus
HCV: hepatitis C virus
US: ultrasonography
CT: computed tomography (CT)
HBM: health belief model
AFP: alpha-fetoprotein
ECOG: Eastern Cooperative Oncology Group
CTP: Child-Turcotte-Pugh
MELD: model for end-stage liver disease
SD: standard deviation
