# Supplementary figures and images for: Oral Medications Enhance Adherence to Surveillance for Hepatocellular Carcinoma and Survival in Chronic Hepatitis B Patients
Source: PLoS One. 2017 Jan 18;12(1):e0166188. doi: 10.1371/journal.pone.0166188 (PMC5242546; doi:10.1371/journal.pone.0166188)

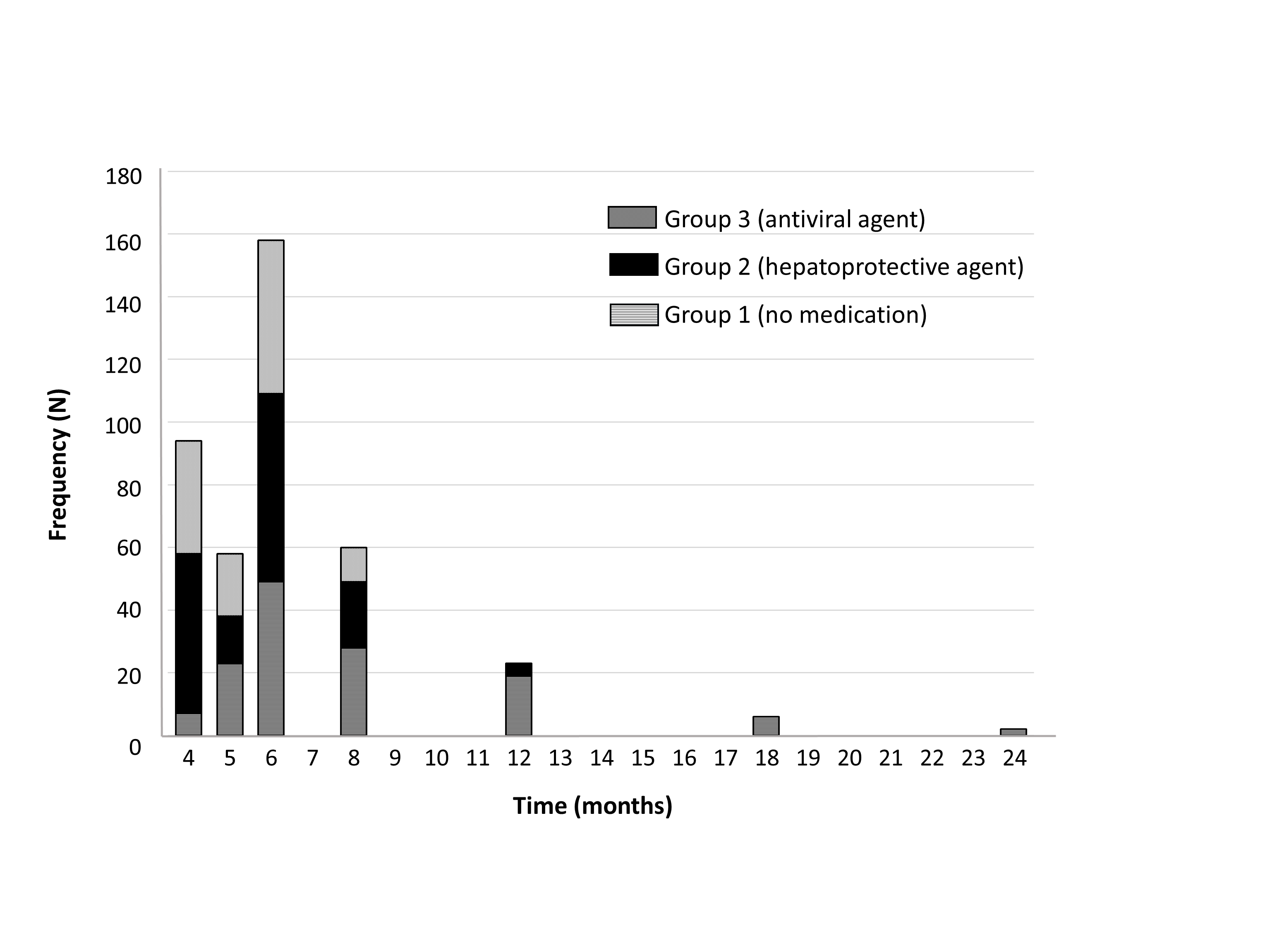

Supplement: S1 Fig — More patients in group 2 (hepatoprotective agents) or 3 (antiviral agent) tended to be followed up at short term imaging interval than patients of group 1 (no medication). Several patients in group 1 had a long term imaging interval which meant poor compliance to surveillance. (TIF) [file pone.0166188.s001.TIF]

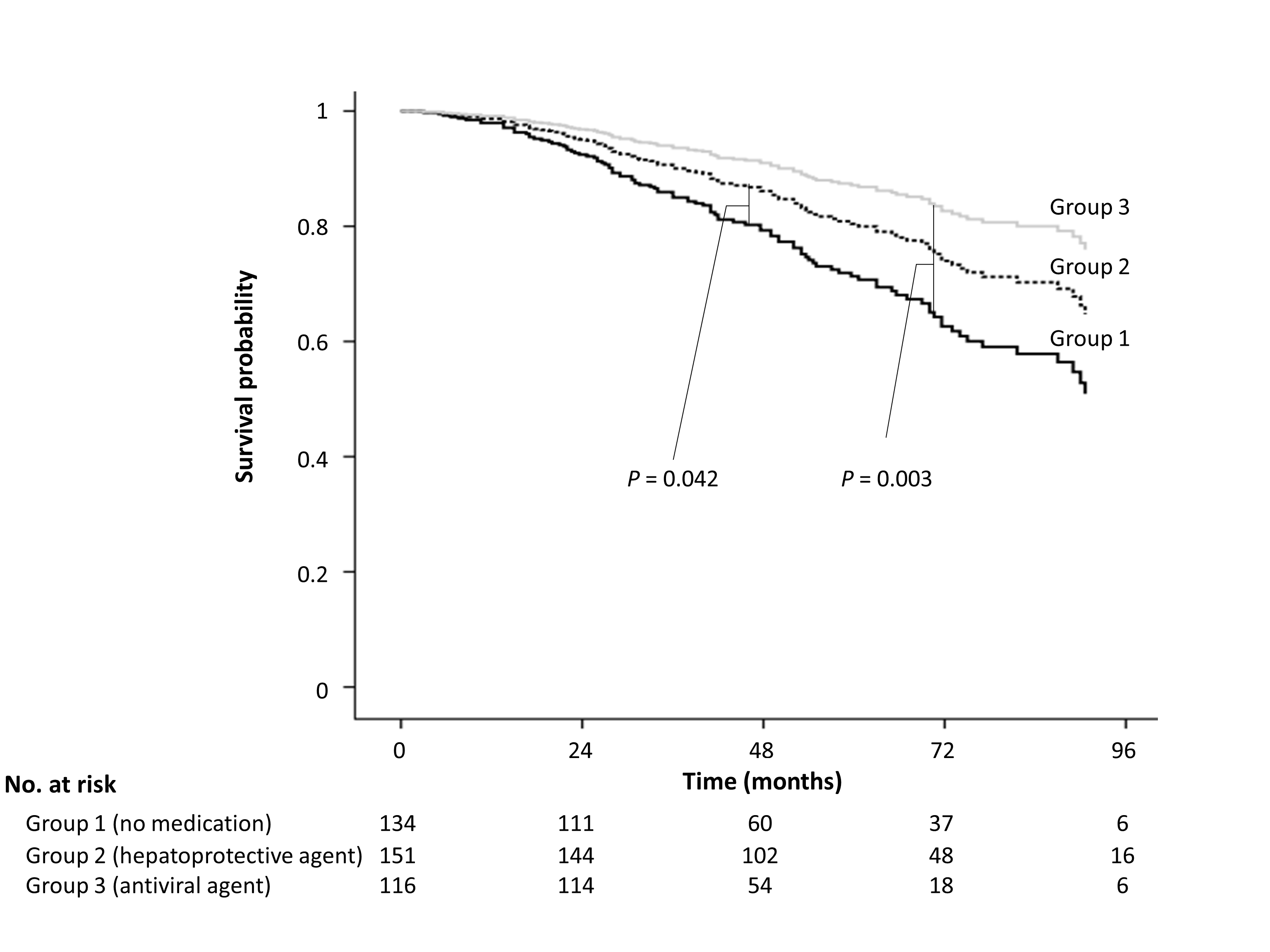

Supplement: S2 Fig — Overall survival was also highest in group 3 (antiviral agents), followed by group 2 (hepatoprotective agents), and OS was lowest in group 1 (no medication) after an adjustment of lead-time bias (all P < 0.05). (TIF) [file pone.0166188.s002.TIF]
