# Supplementary material for: Oral Medications Enhance Adherence to Surveillance for Hepatocellular Carcinoma and Survival in Chronic Hepatitis B Patients
Source: PLoS One. 2017 Jan 18;12(1):e0166188. doi: 10.1371/journal.pone.0166188 (PMC5242546; doi:10.1371/journal.pone.0166188)
Supplement: S1 Table — (DOCX) [file pone.0166188.s003.docx]

**S1 Table. Univariate and multivariate analyses associated with overall survival according to surveillance in no medication group.**

| **Variables** |  | **Univariable analysis** | |  | **Multivariable analysis** | |
| --- | --- | --- | --- | --- | --- | --- |
|  |  | **HR (95% CI)** | ***P*-value** |  | **HR (95% CI)** | ***P*-value** |
| Age |  | 1.02 (0.98-1.06) | 0.335 |  |  |  |
| Sex | Male | 0.62 (0.26-1.47) | 0.275 |  |  |  |
| Cirrhosis |  | 25.62 (0.49-1349.58) | 0.109 |  |  |  |
| ECOG | 0 | 1 (reference) | < 0.001 |  | 1 (reference) |  |
|  | 1 | 2.80 (1.50-5.22) | 0.001 |  | 2.69 (1.37-5.25) | 0.004 |
|  | ≥2 | 20.16 (5.67-71.67) | < 0.001 |  | 42.23 (8.31-214.57) | <0.001 |
| DM |  | 1.74 (0.83-3.65) | 0.870 |  |  |  |
| HTN |  | 1.99 (1.01-3.95) | 0.048 |  | 2.33 (0.82-6.59) | 0.112 |
| Year of HCC | 2007 | 1.03 (0.29-3.72) | 0.962 |  |  |  |
| diagnosis | 2008 | 2.03(0.70-5.94) | 0.194 |  |  |  |
|  | 2009 | 0.76 (0.27-2.12) | 0.603 |  |  |  |
|  | 2010 | 1.25 (0.51-3.06) | 0.320 |  |  |  |
|  | 2011 | 1.22 (0.48-3.16) | 0.646 |  |  |  |
|  | 2012 | 1 (reference) |  |  |  |  |
| Surveillance | Regular | 1 (reference) |  |  | 1 (reference) |  |
|  | Irregular | 1.78 (0.97-3.24) | 0.060 |  | 2.13 (1.07-4.22) | 0.031 |

HR, hazard ratio; CI, confidence interval; ECOG, Eastern Cooperative Oncology Group; DM, diabetes mellitus; HTN, hypertension.

Note. Data are expressed as n (%) or median with minimum and maximum.
