# Supplementary material for: Oral Medications Enhance Adherence to Surveillance for Hepatocellular Carcinoma and Survival in Chronic Hepatitis B Patients
Source: PLoS One. 2017 Jan 18;12(1):e0166188. doi: 10.1371/journal.pone.0166188 (PMC5242546; doi:10.1371/journal.pone.0166188)
Supplement: S2 Table — (DOCX) [file pone.0166188.s004.docx]

**S2 Table. Patients' characteristics at the time of HCC diagnosis according to surveillance within no medication group.**

|  | **Parameter** | **Total (%)** | **Regular** | **Irregular** | ***P-*value** |
| --- | --- | --- | --- | --- | --- |
|  |  | **(*n*=134)** | **(*n*=82)** | **(*n*=52)** |  |
| Age, mean±SD, years |  | 58.0±8.3 | 58.3±8.1 | 57.5±8.7 | 0.600‡ |
| Sex, N (%) | Male | 108 (80.6%) | 68 (82.9%) | 40 (76.9%) | 0.392§ |
| BMI mean±SD, m^2^/kg |  | 23.1±3.1 | 23.1±3.0 | 23.1±3.4 | 0.910§ |
| ECOG^*^, N (%) | 0 | 88 (65.7%) | 55 (67.1%) | 33 (63.5%) | 0.881§ |
|  | 1 | 43 (32.1%) | 25 (30.5%) | 18 (34.6%) |  |
|  | ≥2 | 3 (2.2%) | 2 (2.4%) | 1 (1.9%) |  |
| Diabetes, N (%) |  | 21 (15.7%) | 18 (22.0%) | 3 (5.8%) | 0.012§ |
| Hypertension, N (%) |  | 29 (21.6%) | 21 (25.6%) | 8 (15.4%) | 0.161§ |
| Platelet, ×10^3^/mm^3^ |  | 118.2±41.4 | 119.5±42.8 | 116.0±39.5 | 0.633‡ |
| Albumin, g/dL |  | 3.9±0.5 | 3.9±0.5 | 4.0 ±0.4 | 0.585‡ |
| Total bilirubin, mg/dL |  | 1.5±3.3 | 1.6±4.1 | 1.3±1.2 | 0.562‡ |
| ALP, IU/L |  | 97.6±59.6 | 92.1±58.8 | 106.5±60.4 | 0.173‡ |
| AST, IU/L |  | 48.0±36.0 | 46.6±37.1 | 50.1±34.3 | 0.573‡ |
| ALT, IU/L |  | 48.0±37.3 | 48.3±37.1 | 47.7±27.6 | 0.931‡ |
| PT INR |  | 1.14±0.20 | 1.1±0.2 | 1.2±0.2 | 0.137‡ |
| HBeAg-positive, N (%) |  | 11 (8.2%) | 8 (9.8%) | 3 (5.8%) | 0.755‡ |
| AFP, ng/mL |  | 1320.7±11242.6 | 150.6±641.7 | 3202.2±18087.7 | 0.234‡ |
| MELD score, mean±SD |  | 9.1±3.7 | 9.0±4.2 | 9.3±2.6 | 0.561‡ |
| CTP class, N (%) | A | 116 (86.6%) | 70 (85.4%) | 46 (88.5%) | 0.769§ |
|  | B | 16 (11.9%) | 11 (13.4%) | 5 (9.6%) |  |
|  | C | 2 (1.5%) | 1 (1.2%) | 1 (1.9%) |  |
| Liver cirrhosis^†^, N (%) |  | 122 (91.0%) | 75 (91.5%) | 47 (90.4%) | 0.831§ |
| Year of HCC diagnosis, | 2007 |  | 12 (14.6%) | 5 (9.6%) | 0.055∥ |
| N (%) | 2008 |  | 14 (17.1%) | 8 (15.4%) |  |
|  | 2009 |  | 11 (13.4%) | 12 (23.1%) |  |
|  | 2010 |  | 3 (3.7%) | 8 (15.4%) |  |
|  | 2011 |  | 7 (8.5%) | 6 (11.5%) |  |
|  | 2012 |  | 35 (42.7%) | 13 (25.0%) |  |

BMI, body mass index; ECOG, Eastern Cooperative Oncology Group; ALP, alkaline phosphatase; AST, aspartate transaminase; ALT, alanine transaminase; PT INR, prothrombin time international normalized ratio; AFP, alpha-fetoprotein; MELD, Model for End-stage Liver Disease; CTP, Child-Turcotte-Pugh.

Note. Data are expressed as n (%) or mean±SD.

* The ECOG performance status assesses on a scale ranging from 0 (fully active) to 5 (dead).

† Liver cirrhosis was diagnosed by the presence of histological and radiological evidence.

‡ By Student t-test

§ By Pearson's Chi-square test
∥By Linear-by-linear association test
