# Supplementary material for: Oral Medications Enhance Adherence to Surveillance for Hepatocellular Carcinoma and Survival in Chronic Hepatitis B Patients
Source: PLoS One. 2017 Jan 18;12(1):e0166188. doi: 10.1371/journal.pone.0166188 (PMC5242546; doi:10.1371/journal.pone.0166188)
Supplement: S3 Table — (DOCX) [file pone.0166188.s005.docx]

**S3 Table. Initial tumor characteristics according to surveillance in no mediation group**

| **Tumor** | **Parameter** | **Surveillance** | **Non-surveillance** | ***P*-value** |
| --- | --- | --- | --- | --- |
| **characteristics** |  |  |  |  |
| Type | Nodular | 81 (98.8%) | 49 (94.2%) | 0.132* |
|  | Diffuse/infiltrative/massive | 1 (1.2%) | 3 (5.8%) |  |
| Numbers | Single | 57 (69.5%) | 32 (61.5%) | 0.341* |
|  | Multiple | 25 (30.5%) | 20 (38.5%) |  |
| Maximum size | cm | 1.9±1.1 | 3.1±2.7 | 0.295† |
| MVI |  | 9 (11.0%) | 9 (17.3%) | 0.295* |
| PVTT |  | 10 (12.2%) | 9 (17.3%) | 0.408* |
| BCLC stage | 0 | 20 (24.4%) | 8 (15.4%) | 0.611* |
|  | A | 25 (30.5%) | 15 (28.8%) |  |
|  | B | 4 (4.9%) | 5 (9.6%) |  |
|  | C | 31 (37.8%) | 23 (44.2%) |  |
|  | D | 2 (2.4%) | 1 (1.9%) |  |
| First treatment | Liver transplantation | 1 (1.2%) | 1 (1.9%) | 0.019* |
| modality | Surgical resection | 8 (9.8%) | 10 (19.2%) |  |
|  | RFA | 17 (20.7%) | 8 (15.4%) |  |
|  | PEI | 23 (28.0%) | 2 (3.8%) |  |
|  | TACE | 29 (35.4%) | 27 (51.9%) |  |
|  | Sorafenib | 1 (1.2%) | 1 (1.9%) |  |
|  | Cytotoxic chemotherapy | 0 (0.0%) | 0 (0.0%) |  |
|  | Supportive care only or lost to follow-up | 3 (3.7%) | 3 (5.8%) |  |

MVI, major vessel invasion; PVTT, portal vein tumor thrombosis; BCLC, Barcelona Clinic Liver Cancer; RFA, radiofrequency ablation; PEI, percutaneous ethanol injection; TACE, transarterial chemoembolization.

* By Pearson's Chi-square test

† By Student t-test

‡ By one-way ANOVA
