# Supplementary material for: Oral Medications Enhance Adherence to Surveillance for Hepatocellular Carcinoma and Survival in Chronic Hepatitis B Patients
Source: PLoS One. 2017 Jan 18;12(1):e0166188. doi: 10.1371/journal.pone.0166188 (PMC5242546; doi:10.1371/journal.pone.0166188)
Supplement: S4 Table — (DOCX) [file pone.0166188.s006.docx]

**S4 Table. Results of mediation analysis.**

**(A) Group 1*** **vs. Group 2**†

|  | **Estimates** | **Lower CI** | **Upper CI** | ***P*-value**§ |
| --- | --- | --- | --- | --- |
| Conditional direct effect | 0.980 | 0.675 | 1.424 | 0.916 |
| Direct effect | 0.992 | 0.659 | 1.491 | 0.968 |
| Indirect effect | 1.669 | 1.261 | 2.209 | < 0.001 |
| Total effect | 1.655 | 0.998 | 2.745 | 0.051 |
| Proportion mediated | 1.013 |  |  |  |

**(B) Group 1*** **vs. Group 3**‡

|  | **Estimates** | **Lower CI** | **Upper CI** | ***P*-value**§ |
| --- | --- | --- | --- | --- |
| Conditional direct Effect | 1.737 | 0.826 | 3.651 | 0.145 |
| Direct effect | 1.766 | 0.655 | 4.759 | 0.261 |
| Indirect effect | 1.073 | 0.464 | 2.483 | 0.869 |
| Total effect | 1.896 | 1.166 | 3.082 | < 0.001 |
| Proportion mediated | 0.145 |  |  |  |

CI, confidence interval.

Note.

* Group which is followed with no medication

† Group which is followed with hepatoprotective agents

‡ Group which is followed with antiviral agents

§ By accelerated failure time model
