# Supplementary material for: Oral Medications Enhance Adherence to Surveillance for Hepatocellular Carcinoma and Survival in Chronic Hepatitis B Patients
Source: PLoS One. 2017 Jan 18;12(1):e0166188. doi: 10.1371/journal.pone.0166188 (PMC5242546; doi:10.1371/journal.pone.0166188)
Supplement: S5 Table — (DOCX) [file pone.0166188.s007.docx]

**S5 Table. Univariate and multivariate analyses associated with overall survival according to median follow-up interval.**

| **Variables** |  | **Univariable analysis** | |  | **Multivariable analysis** | |
| --- | --- | --- | --- | --- | --- | --- |
|  |  | **HR (95% CI)** | ***P*-value** |  | **HR (95% CI)** | ***P*-value** |
| Age |  | 1.018 (0.99-1.04) | 0.147 |  |  |  |
| Sex | Male | 1.543 (0.92-2.60) | 0.103 |  |  |  |
| Cirrhosis |  | 17.63 (2.46-126.54) | 0.004 |  | 15.43 (2.15-111.00) | 0.007 |
| ECOG | 0 | 1 (reference) | < 0.001 |  | 1 (reference) |  |
|  | 1 | 2.19 (1.47-3.260) |  |  | 1.91 (1.28-2.85) | 0.002 |
|  | ≥2 | 9.16 (3.86-21.76) |  |  | 9.70 (4.05-23.21) | <0.001 |
| DM |  | 0.98(0.57-1.61) | 0.870 |  |  |  |
| HTN |  | 1.26 (0.78-2.04) | 0.352 |  |  |  |
| Year of HCC | 2007 | 0.66 (0.24-1.81) | 0.416 |  |  |  |
| diagnosis | 2008 | 1.10(0.52-2.33) | 0.807 |  |  |  |
|  | 2009 | 1.30 (0.66-2.55) | 0.460 |  |  |  |
|  | 2010 | 1.47 (0.75-2.91) | 0.264 |  |  |  |
|  | 2011 | 1.31 (0.66-2.58) | 0.439 |  |  |  |
|  | 2012 | 1 (reference) |  |  |  |  |
| Follow-up | ≤ 6 months | 1 (reference) |  |  | 1 (reference) |  |
| interval | > 6 months | 2.69 (1.81-4.01) | <0.001 |  | 2.63 ( 1.75-3.96) | <0.001 |

HR, hazard ratio; CI, confidence interval; ECOG, Eastern Cooperative Oncology Group; DM, diabetes mellitus; HTN, hypertension.

Note. Data are expressed as n (%) or median with minimum and maximum.
