# Supplementary material for: Oral Medications Enhance Adherence to Surveillance for Hepatocellular Carcinoma and Survival in Chronic Hepatitis B Patients
Source: PLoS One. 2017 Jan 18;12(1):e0166188. doi: 10.1371/journal.pone.0166188 (PMC5242546; doi:10.1371/journal.pone.0166188)
Supplement: S6 Table — (DOCX) [file pone.0166188.s008.docx]

**S6 Table. Univariate and multivariate analyses associated with overall survival after adjustment for lead-time bias.**

| **Variables** |  | **Univariable analysis** | |  | **Multivariable analysis** | |
| --- | --- | --- | --- | --- | --- | --- |
|  |  | **HR (95% CI)** | ***P*-value** |  | **HR (95% CI)** | ***P*-value** |
| Age |  | 1.018 (0.99-1.04) | 0.147 |  |  |  |
| Sex | Male | 1.558 (0.93-2.62) | 0.095 |  |  |  |
| Cirrhosis |  | 17.74 (2.47-127.3) | 0.004 |  | 15.88 (2.21-114.15) | 0.006 |
| ECOG | 0 | 1 (reference) | < 0.001 |  | 1 (reference) |  |
|  | 1 | 2.16 (1.45-3.21) |  |  | 2.08 (1.40-3.10) | < 0.001 |
|  | ≥2 | 9.68 (4.07-23.05) |  |  | 8.55 (3.59-20.34) | < 0.001 |
| DM |  | 0.89(0.57-1.62) | 0.889 |  |  |  |
| HTN |  | 1.27 (0.78-2.05) | 0.334 |  |  |  |
| Year of HCC | 2007 | 0.67 (0.24-1.83) | 0.431 |  |  |  |
| diagnosis | 2008 | 1.10 (0.52-2.33) | 0.799 |  |  |  |
|  | 2009 | 1.28 (0.65-2.52) | 0.479 |  |  |  |
|  | 2010 | 1.44 (0.73-2.85) | 0.294 |  |  |  |
|  | 2011 | 1.28 (0.65-2.53) | 0.472 |  |  |  |
|  | 2012 | 1 (reference) |  |  |  |  |
| Medication | Group 1* | 1 (reference) |  |  | 1 (reference) |  |
|  | Group 2† | 0.74 (0.48-1.12) | 0.124 |  | 0.64 (0.42-0.98) | 0.042 |
|  | Group 3‡ | 0.43 (0.24-0.77) | 0.004 |  | 0.41 (0.23-0.73) | 0.003 |

HR, hazard ratio; CI, confidence interval; ECOG, Eastern Cooperative Oncology Group; DM, diabetes mellitus; HTN, hypertension.

Note. Data are expressed as n (%) or median with minimum and maximum.

* Group which is followed with no medication

† Group which is followed with hepatoprotective agents

‡ Group which is followed with antiviral agents
